# Organic Germanium (Ge-132) Reduces Glycative Damage While Maintaining Cellular Stress Signaling, Revealing Limited Coordination Between Biochemical and Cellular Responses

**DOI:** 10.3390/molecules31142405

**Published:** 2026-07-08

**Authors:** Yasin Fauzi Ahmed Elhdiri, Gabrielle Guillaumin, Amanda Martell Vergara, Lucia Gimeno Mallech, Antonella Locascio

**Affiliations:** School of Health Sciences, Universidad Cardenal Herrera-CEU, CEU Universities, Calle Santiago Ramón y Cajal 20, Alfara del Patriarca, 45115 Valencia, Spain; yasin.fauzielhdiri@alumnos.uchceu.es (Y.F.A.E.); gabrielle.guillaumin@alumnos.uchceu.es (G.G.); amanda.martell@alumnos.uchceu.es (A.M.V.); lucia.gimenomallench@uchceu.es (L.G.M.)

**Keywords:** organic germanium, oxidative stress, glycation, inflammation, AGEs

## Abstract

Organogermanium compounds, particularly carboxyethyl germanium sesquioxide (Ge-132), have been studied for decades because of their diverse biological effects, especially their antioxidant properties. However, the available literature remains fragmented and highly heterogeneous, which limits mechanistic interpretation. Although antiglycative activity has been described at the biochemical level, the downstream transcriptional effects of Ge-132 under glycative stress remain poorly characterized. Here, we combined an evidence-mapping analysis with targeted molecular analysis in a standardized cellular model to examine whether the antiglycative effects of Ge-132 are accompanied by coordinated transcriptional responses. The mapping guided selection of markers associated with glycative stress, including carbonyl detoxification, redox adaptation, autophagy, lysosomal function, and inflammatory signaling. Gene expression analysis revealed limited and selective transcriptional modulation under glycative stress conditions. In parallel, protein analysis showed reduced intracellular accumulation of advanced glycation end products (AGEs) in Ge-132–treated. These findings suggest that reduction of glycative damage can occur without proportional transcriptional activation of stress-response pathways under the conditions tested. However, alternative explanations, such as model constraints, acute exposure duration, or limited gene panel sensitivity, cannot be excluded.

## 1. Introduction

Germanium (Ge) is a naturally occurring trace element found in soils, rocks, and several plants, including garlic, ginseng, aloe, shiitake mushrooms, and pearl barley [1,2]. It has physicochemical properties intermediate between metals and non-metals, supporting diverse technological and biomedical applications [1,2]. Although initially used in electronics, interest in the biological properties of germanium increased during the 1970s–1980s. Germanium compounds are classified as inorganic or organic, differing in physicochemical and biological properties. Inorganic forms are poorly soluble, accumulate in organs, and can cause dose-dependent, often irreversible toxicity, including nephrotoxicity and neurotoxicity [1,3,4,5,6,7]. Nevertheless, germanium-68 (^68^Ge) is used in nuclear medicine as a parent radionuclide for gallium-68 (^68^Ga) in PET imaging for cancer and inflammation detection [8,9,10].

Organogermanium compounds exhibit distinct biological and toxicological profiles. The best-characterized compound is Ge-132 (Repagermanium), a water-soluble polymer that hydrolyzes into 3-(Trihydroxygermyl) propanoic acid (THGP). Compared with inorganic forms, Ge-132 shows low toxicity and minimal tissue accumulation [3,5,11,12,13,14,15]. THGP forms complexes with cis-diol-containing biomolecules, including saccharides and nucleotides. This interaction is thought to underlie its biological effects, particularly the modulation of oxidative stress [2,16,17].

Ge-132 has been associated with beneficial effects on mitochondrial metabolism, immune signaling, and stress adaptation. Notably, it does not appear to act as a direct ROS scavenger. Instead, it may modulate oxidative stress indirectly by regulating mitochondrial metabolism and endogenous antioxidant systems, thereby supporting redox homeostasis [7,18,19]. THGP has been reported to preserve ATP levels, enhance oxidative phosphorylation, and reduce ROS and lipid peroxidation. Proposed mechanisms include complex formation with cis-diol- containing biomolecules, inhibition of adenosine deaminase, stimulation of aldehyde reductase, and suppression of AGEs formation [1,7,19,20]. Ge-132 has also been shown to modulate transcriptional responses, including suppression of *NR4A2* and pro-inflammatory mediators such as *IL-6* and *CXCL2*, as well as regulation of apoptosis-related pathways involving KEAP1–BAX–caspase-3 and BCL-2 [21]. However, the high variability in experimental models and conditions limits direct comparison across studies and complicates mechanistic interpretation.

Ge-132 also influences immune and inflammatory responses, enhancing IFN-γ production, NK cell activity, and B-cell responses [17,22,23,24,25]. For instance, THGP regulates inflammation by sequestering extracellular ATP and inhibiting caspase-1–mediated IL-1β release [22,26]. It also promotes tissue repair via immune cell recruitment and *TGF-β*–mediated collagen synthesis [27], and preservation of bone integrity [1,7,19,28].

Antitumor effects have also been reported, including reduced tumor viability, inhibition of metastasis, modulation of SIRP-α–CD47 signaling, and induction of cell-cycle arrest and apoptosis [1,20,22,23,29,30]. Clinical observations suggest additional supportive effects in palliative care [22,31].

Similar protective roles have been observed in plants, where germanium enhances growth and salt stress tolerance via oxidative balance modulation [32,33,34,35,36].

In in vivo and cellular models, Ge-132 also affects aging-related pathways by supporting antioxidant defenses, immune function, and cell renewal. It has been documented that THGP enhances clearance of senescent Red Blood Cells and reduces oxidative stress–induced apoptosis, with associated modulation of *Nrf-2* and *Bax* genes, while preserving mitochondrial function and ATP levels [1,20,37,38]. The antioxidant properties of Ge-132 and THGP extend to the modulation of glycative stress. Rather than reflecting a classical antioxidant mechanism, this effect appears to be mediated by complex formation with cis-diol–containing monosaccharides, thereby interfering with the Maillard reaction and reducing the formation of AGEs [39,40,41,42,43]. This antiglycative activity has been demonstrated in diabetic experimental models, where protein glycation is significantly inhibited [38,44,45,46]. In contrast to the relatively well-described biochemical antiglycative effects, the downstream gene regulatory responses associated with glycative stress under Ge-132 exposure remain largely unexplored. This gap limits current mechanistic understanding, particularly considering that AGEs formation is a major source of reactive oxygen species and that its inhibition may indirectly contribute to the regulation of oxidative stress and maintenance of cellular homeostasis. In addition to the biological effects described above, the intestinal microbiota may represent another level at which dietary Ge-132 influences host homeostasis. Although Ge-132 does not appear to markedly alter caecal microbial composition, it interacts functionally with the microbial ecosystem, particularly in the presence of prebiotics or probiotics [16,25,26]. In combination with oligosaccharides such as raffinose and with *Lactobacillus* species, Ge-132 enhances mucosal immune responses [20,25]. Interestingly, Ge-132 alone has been reported to increase β-glucuronidase activity, a marker associated with elevated colon cancer risk, although this effect is abolished when prebiotics are co-administered, underscoring the context-dependent biological effects of Ge-132 [47].

Together, these observations suggest that organic Germanium influences multiple biological processes, including redox regulation, immune signaling, metabolic adaptation, and stress resilience. However, the available evidence originates from heterogeneous experimental systems and addresses distinct biological endpoints, resulting in a fragmented body of literature that limits mechanistic interpretation and precludes meaningful quantitative synthesis.

Rather than performing a quantitative synthesis, the present study adopted an evidence-informed experimental approach. The evidence-mapping component was designed to identify underexplored areas and methodological gaps in the Ge-132 literature, rather than to establish definitive mechanistic relationships. The analysis highlighted glycative stress as a relatively underexplored research area, despite the well-documented antiglycative properties of Ge-132. Importantly, the literature review did not identify any studies that simultaneously evaluated the antiglycative effects of Ge-132 together with downstream cellular responses within a single standardized experimental framework. Reductions in AGE accumulation have been attributed to multiple, not always convergent mechanisms across heterogeneous experimental systems [16,17,23,25,26,30,39,40,41,42,43,44,45,46], making it difficult to determine whether these biochemical effects are consistently linked to coordinated cellular responses.

**Figure 1:**
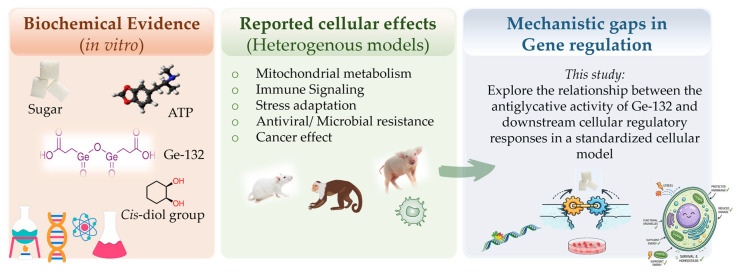
Conceptual overview of current knowledge on organogermanium compounds. Biochemical studies indicate that Ge-132 interacts with cis-diol–containing molecules, including sugars and ATP-related metabolites. Cellular studies across heterogeneous model systems report effects on multiple stress-response pathways. However, the molecular mechanisms linking these biochemical interactions to downstream transcriptional responses remain unclear. In the present study, glycative stress was used as a standardized experimental framework to examine whether the antiglycative effects of Ge-132 are accompanied by coordinated transcriptional responses under controlled conditions.

These observations directly informed the experimental design. Glycative stress was selected as the biological context, and the transcriptional markers examined—genes associated with carbonyl detoxification, redox adaptation, autophagy initiation, lysosomal function, and inflammatory signaling—were chosen because these pathways have been repeatedly implicated in Ge-132 studies, yet their coordinated transcriptional responses had not been investigated together under glycative stress conditions (Figure 1).

## 2. Results

### 2.1. Evidence-Mapping of the Literature

#### 2.1.1. Screening and Selection

**Figure 2:**
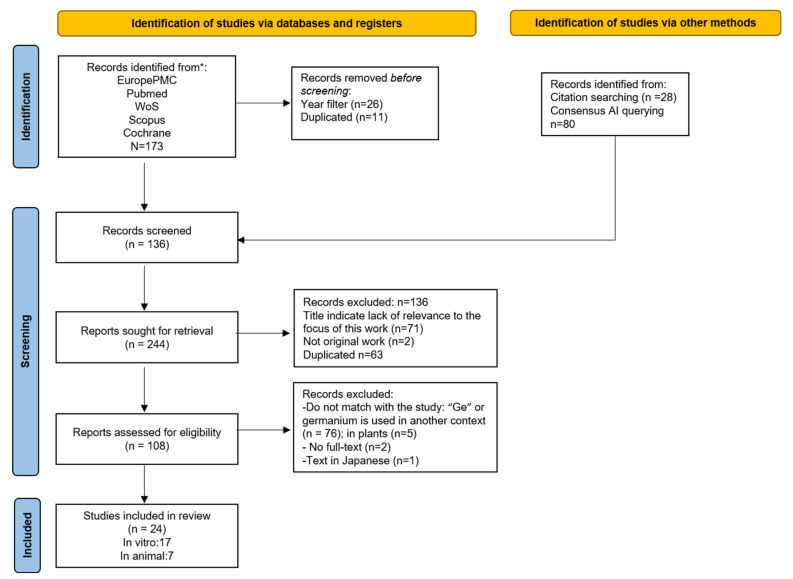
PRISMA flow diagram showing the selection process for studies published in the last 15 years.

The literature search was carried out between September 2025 and January 2026. The screening process is summarized in Figure 2. After removal of duplicates and application of predefined inclusion criteria, 24 studies investigating the biological effects of organic germanium (Ge-132) were selected for qualitative evaluation. This dataset represents a subset of currently available experimental evidence based on the applied criteria addressing the biological activity of Ge-132.

**Figure 3:**
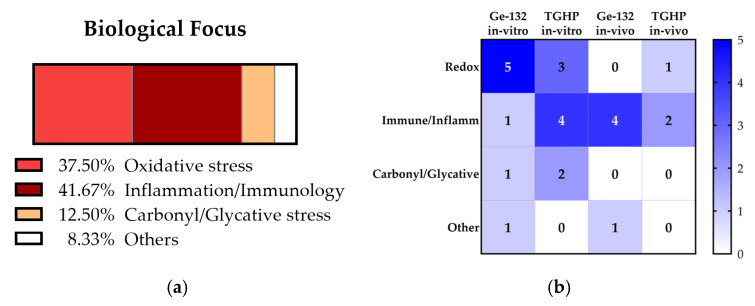
Evidence mapping. (**a**) Distribution of experimental studies on the biological activity of organogermanium compounds by area of investigation, over the past 15 years. (**b**) Distribution of experimental evidence across experimental models (in vivo, in vitro), compounds tested (Ge-132 or THGP) and outcome domains (redox, immune/inflammatory, glycative stress, and others). Color intensity indicates the number of studies per category.

The approach followed does not constitute a formal systematic review or meta-analysis but rather an evidence-informed mapping strategy designed to guide experimental design. The studies were evaluated by comparing those conducted exclusively in vitro with those performed in in vivo models. Only a small number of studies combined both in vitro and in vivo approaches. The analysis of evidence identified three main biological axes: redox regulation, immune/inflammatory signaling, and glycative/carbonyl stress (Figure 3a). Among those, redox and immune-related pathways were the most frequently investigated, whereas glycative stress was comparatively underrepresented. This distribution indicates that, although multiple biological effects have been attributed to Ge-132, research efforts have predominantly focused on classical stress and immune pathways, while glycative stress, despite its relevance to metabolic and age-related processes, remains insufficiently explored.

Evidence mapping integrating compound type, experimental model, and outcome domain confirmed this imbalance (Figure 3b). A predominance of in vitro studies and redox-related outcomes was observed across both Ge-132 and THGP. In contrast, glycative stress studies were limited and mainly restricted to in vitro systems.

Overall, these observations highlight a scattered evidence base, with limited mechanistic continuity and a lack of studies addressing gene regulatory responses under glycative stress conditions.

#### 2.1.2. Implications for Quantitative Revision

The included studies span a broad range of biological processes, such as redox regulation, immune signaling, apoptosis, and metabolic modulation, but show substantial heterogeneity in experimental models, compound forms, dosing strategies, and outcome measures (Table 1 and Table 2 and Table S1), precluding meaningful quantitative synthesis. Beyond this methodological limitation, the mapping identified a specific content gap: glycative stress remains comparatively underexplored in the Ge-132 literature, and no available study had examined transcriptional responses associated with carbonyl detoxification, redox adaptation, autophagy initiation, lysosomal function, and inflammatory signaling together within a single experimental framework under glycative stress conditions. The methodological fragmentation and the specific content gap jointly motivated the experimental component of the present study.

### 2.2. Risk of Bias Assessment

**Figure 4:**
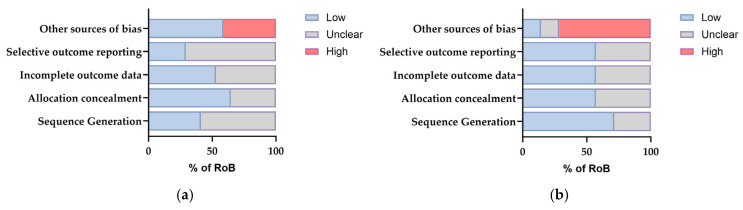
Risk-of-bias assessment of methodological reliability across quality domains. Data represent the percentage of risk in the total number of publications included in each sample category. (**a**) In vitro based research; (**b**) In vivo based research.

To evaluate methodological reliability, each study was assessed using an adapted CASP risk-of-bias framework [54]. Although the CASP tool was originally developed for clinical evidence, an adapted version was applied here to provide a structured qualitative appraisal of heterogeneous preclinical studies. This approach aligns with common practices in narrative and evidence-mapping reviews of emerging compounds. In the case of in vitro studies (Figure 4a), the RoB assessment revealed moderate methodological variability. Sequence generation procedures were frequently insufficiently reported, resulting in a substantial proportion of unclear risk classifications, whereas allocation of concealment was generally well described. Incomplete outcome data showed a balanced distribution between low and unclear risk, suggesting acceptable reporting despite limited documentation of exclusions. Notably, selective outcome reporting was predominantly classified as unclear, indicating that experimental endpoints were often not predefined or fully described. Additionally, the presence of high-risk classifications within the “Other sources of bias” domain primarily reflects the involvement of research groups associated with the development or provision of the organogermanium compound under investigation. While such collaborations may facilitate compound standardization and experimental consistency, the limited independent replication across laboratories may introduce potential sponsorship-related bias.

The RoB assessment for in vivo studies (Figure 4b) indicated a generally adequate experimental structure, with low risk predominating in the domains of sequence generation and incomplete outcome data. Allocation of concealment and selective outcome reporting were frequently classified as unclear due to incomplete methodological reporting. The “Other sources of bias” domain showed a predominance of high-risk classifications, reflecting limited independent replication across research groups and potential structural influences related to compound sourcing.

Overall, the comparison between in vitro and in vivo studies highlights substantial variability in methodological reporting and experimental design across the Ge-132 literature. This underscores the need for more standardized experimental frameworks to enable meaningful comparisons across studies.

Another notable observation emerging from the literature analysis is the limited continuity between research lines. Most studies consist of isolated investigations that are rarely followed by subsequent work exploring the same biological processes in depth or across complementary models.

Although some thematic progression is evident within specific research groups, particularly among those from Asian institutes, these efforts often shift across different biological contexts rather than systematically building upon a common framework. This pattern contributes to the fragmentation of the field and limits the development of robust, reproducible evidence.

### 2.3. Cytotoxicity Assay Across Distinct Cell Lines

Three cellular models were initially evaluated for Ge-132 cytotoxicity and differential sensitivity: human lens epithelial cells (HLECs), human retinal pigment epithelial cells (ARPE-19), and mouse embryonic fibroblasts (MEFs). MEFs were included as a well-characterized model commonly used to study cellular responses to glycative stress, oxidative stress, proteostasis disruption, and autophagy-related processes [55,56]. The concentration range of Ge-132 was selected based on the doses most frequently reported in the literature (Table 1).

**Figure 5:**
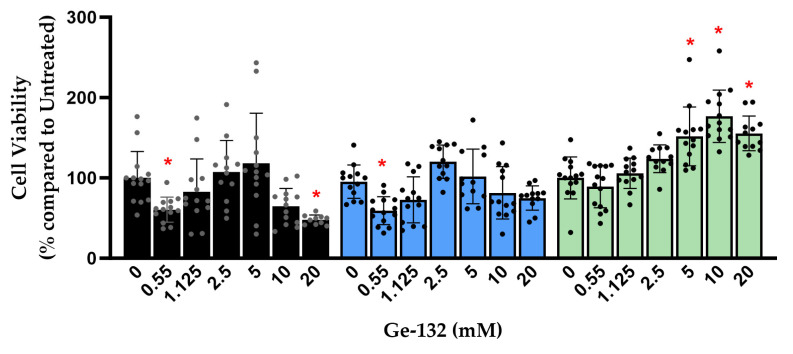
Cytotoxicity assessment of Ge-132 across different cell lines. Cell viability was assessed in MEFs (black bars), HLECs (blue-bars), and ARPE-19 cells (green-bars) following exposure to increasing concentrations of Ge-132. Data represent the mean of five biological replicates (10 to 15 technical replicates each) ± SD. Significant differences relative to the corresponding control were marked with a red asterisk (*), after one-way ANOVA followed by Dunnett’s multiple comparison test.

In general, Ge-132 did not induce significant cytotoxicity in HLECs or ARPE-19 cells within the concentration range tested. In contrast, MEFs displayed greater sensitivity to Ge-132 at higher concentrations (Figure 5). Based on these results, MEFs were selected for subsequent cellular response analyses. The use of a single cellular model enabled direct comparison of multiple biological pathways within the same experimental framework while minimizing variability associated with cross-model analyses, thereby facilitating interpretation of transcriptional responses across pathways.

### 2.4. Antiglycative Activity of Ge-132

**Figure 6:**
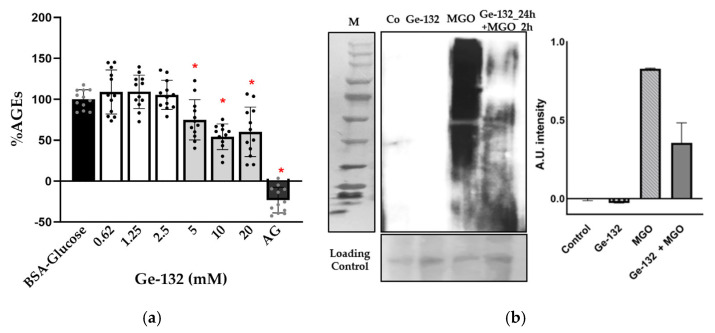
Antiglycative activity of Ge-132 in cell-free and cellular systems. (**a**) In vitro Maillard reaction assay showing dose-dependent reduction of AGE formation by Ge-132 (5–20 mM). AGE levels are expressed relative to the positive control (BSA + Glucose). Aminoguanidine (AG) was used as a reference inhibitor. The graph is representative of three independent experiments, each with 12 technical replicates. Data were analyzed by one-way ANOVA followed by Dunnett’s post hoc test. The error bar represents SD. (**b**) Immunodetection of methylglyoxal-derived protein adducts in MEF cells exposed to MGO, with or without 5 mM Ge-132 pre-treatment. Anti-MG was used to detect MGO-derived protein adducts. Ponceau S staining served as a loading and transfer control. Densitometric quantification of the immunoblot signal is shown. Red asterisks (*) indicate statistically significant differences determined by Student’s *t*-test (*p* < 0.05), respect to BSA-Glucose control.

Based on the gaps identified in the literature mapping, we focused our experimental analysis on transcriptional responses associated with glycative stress. To evaluate the antiglycative activity of Ge-132 across different levels of biological complexity, we first assessed its effect in a cell-free Maillard reaction system using a concentrations range of 5–20 mM. Based on cytotoxicity assessment and literature-reported concentrations, subsequent cellular experiments (qPCR and Western blot) were performed using 5 mM Ge-132. Under these conditions, Ge-132 reduced the formation of glycation products in a dose-dependent manner, with effects detectable from 5 mM onwards (Figure 6). These results are consistent with previously reported antiglycative activity of Ge-132 and demonstrate its ability to reduce AGE accumulation even under glycative stress conditions.

We next investigated whether this effect could be reproduced in a cellular context. Mouse embryonic fibroblasts (MEFs) were exposed to glycative stress induced by methylglyoxal (MGO), either with or without Ge-132. In addition to co-treatment, and consistent with previously reported short-term glycative stress models [57], a sequential protocol was included in which cells were pre-treated with Ge-132 followed by exposure to MGO for 2 h. This condition was designed to explore potential preventive or modulatory effects of Ge-132 against glycative insult. Western blot analysis confirmed the accumulation of AGE-modified proteins under MGO treatment, consistent with the induction of glycative stress conditions. Ge-132 significantly reduced intracellular AGE accumulation in MGO-exposed fibroblasts, with a stronger effect when administered as pre-treatment. Importantly, Ge-132 alone did not alter AGE levels compared with the untreated control, indicating that its antiglycative effect is conditional upon stress exposure rather than a constitutive inhibition of glycation (Figure 6b). These findings demonstrate that the anti-glycative activity of Ge-132 previously observed in cell-free systems can be reproduced in a cellular context, supporting its ability to modulate glycative stress under biologically relevant conditions.

### 2.5. Gene Expression Analysis

To investigate whether the antiglycative effects observed at the biochemical level and confirmed by Western blot analysis are accompanied by measurable cellular transcriptional responses, we analyzed the expression of selected regulatory genes involved in pathways linked to glycative stress. Target genes were chosen based on their established roles as key regulatory nodes in glycative stress response pathways [58,59,60,61,62,63].

#### 2.5.1. Antioxidant Response

Multiple in vitro studies have described antioxidant-like effects of Ge-132 without evidence of strong direct ROS scavenging, instead suggesting indirect modulation of redox signaling pathways (see Table 1 and Table 2). Animal studies have similarly reported reduced oxidative damage and improved physiological outcomes following Ge-132 administration.

**Figure 7:**
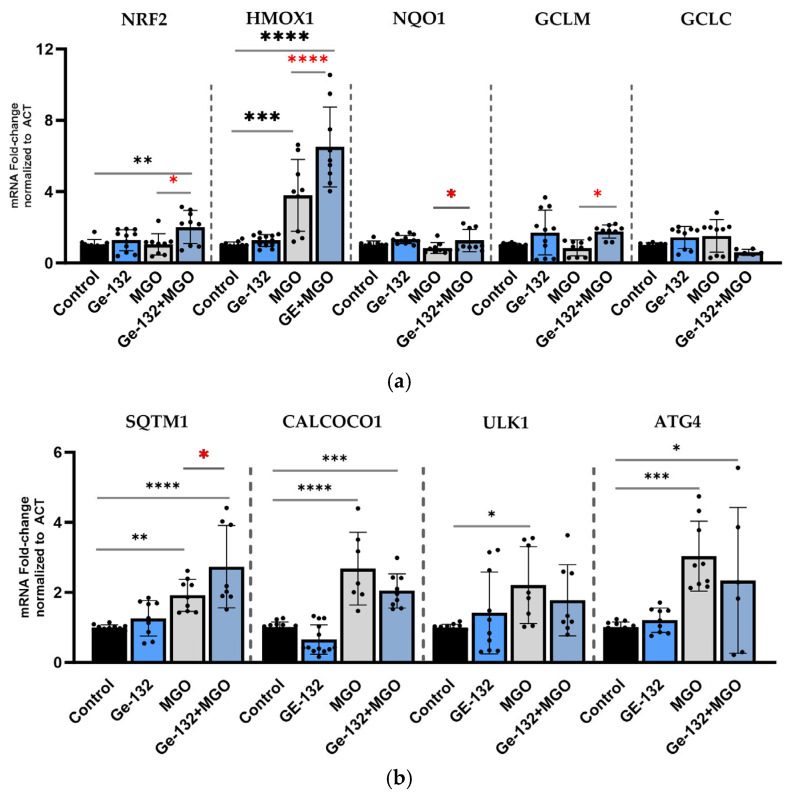
Transcriptional response of antioxidant and autophagy-related genes under glycative stress and Ge-132 treatment. (**a**) Relative expression of *NRF2* and *NRF2*-related genes (*HMOX1*, *NQO1*, *GCLC*, and *GCLM*). (**b**) Relative expression of autophagy-related genes (*SQSTM1, CALCOCO1*, *ULK1* and *ATG4*). Data were normalized to actin and are presented as mean ± SD of three biological replicates. Statistical analysis was performed using one-way ANOVA followed by Dunnett’s post hoc test. Additional pairwise comparisons were evaluated using Student’s *t*-test and are indicated with a red asterisk (*). Statistical significance is indicated as follows: * *p* < 0.05, ** *p* < 0.01, *** *p* < 0.001, and **** *p* < 0.0001.

To evaluate the impact of glycative stress on oxidative stress–related transcriptional responses, we analyzed the expression of *NRF2,* a master regulator of this pathway, along with its downstream targets *HMOX1* and *NQO1*, which are central to redox adaptation (Figure 7a). We also examined *GCLC* and *GCLM*, genes involved in glutathione synthesis.

MGO alone did not significantly induce *NRF2* transcription. However, combined Ge-132 + MGO treatment produced a modest but consistent increase in *NRF2* expression. *NQO1* showed a numerical increase under the combined treatment Ge-132 + MGO but did not remain statistically significant after correction for multiple comparisons and should therefore be interpreted cautiously. *HMOX1* was significantly upregulated, consistent with activation of oxidative stress–related transcriptional responses. Although *HMOX1* is a canonical *NRF2* target, it is also regulated by other stress-responsive transcription factors, including *NF-κB*, which can drive its induction under inflammatory or oxidative conditions [64].

Ge-132 alone did not significantly alter the expression of these key antioxidant genes. In contrast, the combination of Ge-132 and MGO induced both *NRF2* and *HMOX1*, indicating partial persistence of oxidative stress–related transcriptional signaling under these conditions. *GCLC* and *GCLM* remained largely unchanged across experimental conditions, further supporting selective rather than broad transcriptional activation.

Although transcript levels do not always correlate with protein abundance or activity, the MGO-induced increase in *NRF2* and *HMOX1* expression in the presence of Ge-132 is consistent with oxidative stress-related transcriptional responses. Collectively, these findings suggest that Ge-132 may reduce glycative damage primarily through upstream biochemical interactions while preserving selective stress-adaptive transcriptional responses under the tested conditions.

#### 2.5.2. Autophagy

Inhibition of carbonyl stress and AGE-associated accumulation has emerged as a key biological outcome of Ge-132 exposure [16,41,42,43,44,45,46,47]. Previous studies have reported improved cellular resilience and reduced stress-induced damage in several in vitro models, including dermal fibroblasts and reproductive systems, indirectly suggesting enhanced handling of damaged biomolecules [18,19,20,21,28,37,48]. Autophagy is the primary cellular process responsible for clearing toxic intracellular accumulations [55].

To determine whether Ge-132-mediated modulation of glycative stress is associated with transcriptional changes in autophagy-related pathways, we examined genes involved in cargo recognition, pathway initiation, and autophagosome formation. These included *SQSTM1*, *CALCOCO1*, *ULK1*, and several *ATG*-related genes as representative markers of selective autophagy and early autophagy signaling (Figure 7b and Figure S1).

MGO exposure induced a slight but significant increase in *SQSTM1* and *CALCOCO1* expression. These genes encode cargo-recognition receptors that label damaged proteins and organelles for degradation, indicating transcriptional modulation of selective autophagy-related processes under glycative stress. *SQSTM1* expression was further elevated under combined Ge-132 + MGO treatment, while *CALCOCO1* levels remained similar to those with MGO alone.

At the level of pathway initiation, *ULK1* and *ATG4* were upregulated by MGO. ULK1 regulates autophagy initiation, and ATG4 is involved in LC3 processing, consistent with transcriptional modulation of early autophagy signaling. The presence of Ge-132 did not significantly attenuate this induction. Genes involved in autophagosome elongation and membrane expansion (*ATG2B*, *ATG3*, *ATG5*, and *ATG7)* showed no significant variation across experimental conditions, indicating that transcriptional modulation primarily affects the early stages of the autophagy pathway (Supplementary Figure S1).

#### 2.5.3. Glyoxalase System and Lysosomal Pathway

**Figure 8:**
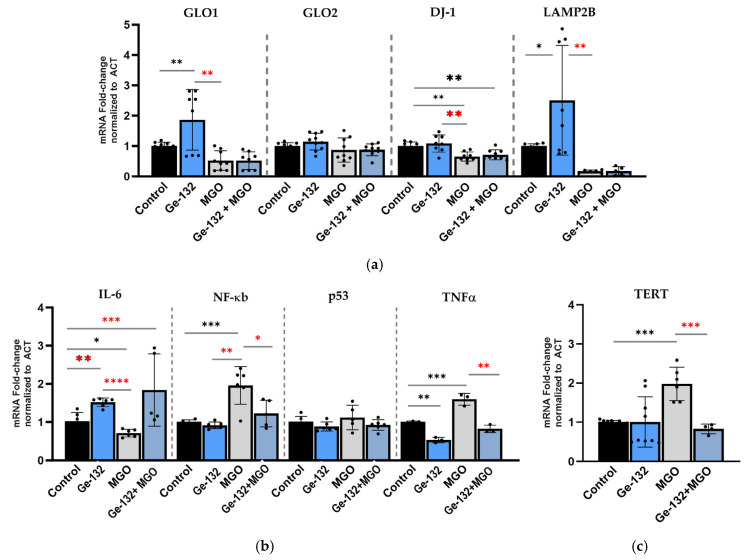
Transcriptional modulation of detoxification, lysosomal, and inflammatory pathways. (**a**) Expression of glyoxalase-related genes (*GLO1*, *GLO2*, *DJ-1*) and the lysosomal marker *LAMP2B*. (**b**) Expression of inflammatory markers (*IL-6*, *NF-κB*, *p53*, and *TNF-α*). (**c**) Expression of *TERT*. Data were normalized to β-actin and are presented as mean ± SD from three biological replicates. Statistical analyses were performed using one-way ANOVA followed by Dunnett’s post hoc test. Additional pairwise comparisons were evaluated using Student’s *t*-test and are indicated in red asterisk (*). Statistical significance is indicated as follows: * *p* < 0.05, ** *p* < 0.01, *** *p* < 0.001, and **** *p* < 0.0001.

To further evaluate detoxification and degradation capacity under glycative stress and the effect of Ge-132 on these processes, we analyzed the expression of key components of the glyoxalase system and lysosomal-associated genes, such as *GLO1*, *GLO2*, and *DJ-1* (Figure 8a).

*GLO1* expression increased under Ge-132 treatment alone, whereas MGO exposure tended to reduce *GLO1* level, although this reduction was not consistently significant after correction for multiple comparisons. As the primary enzyme responsible for methylglyoxal detoxification and for limiting AGE formation [65,66], GLO1 induction by Ge-132 may reflect transcriptional support for carbonyl detoxification pathways. *GLO2* expression remained unchanged across all experimental conditions.

*DJ-1* expression decreased under MGO exposure and was not restored by Ge-132 pre-treatment. *DJ-1* is a redox-sensitive protein involved in antioxidant defense and the detoxification of reactive carbonyl species [67,68]. Its persistent downregulation even in the presence of Ge-132 suggests that the observed reduction in AGEs accumulation is unlikely to result from transcriptional restoration of intracellular detoxification pathways under these experimental conditions.

Among lysosomal genes, *LAMP2B* expression was significantly upregulated by Ge-132 alone. However, MGO exposure caused a pronounced reduction in *LAMP2B* levels that persisted in the combined Ge-132 + MGO condition. *LAMP2B* is associated with lysosomal function and autophagy-related degradation processes [69]. In contrast, *LAMP2A/C* expression showed no significant changes across treatments (Supplementary Figure S1).

Overall, these results indicate that glycative stress downregulates key detoxification and lysosomal components, while Ge-132 primarily influences these pathways under non-stressed conditions.

#### 2.5.4. Inflammatory Response

Animal studies have reported immunomodulatory properties of organogermanium compounds, including suppression of inflammasome activation, modulation of antiviral sensing pathways [51], and enhanced macrophage-mediated phagocytic activity [22]. Additional in vivo work has shown reduced inflammatory damage in various disease models [50].

To assess whether glycative stress and its modulation by Ge-132 influence inflammatory signaling, we analyzed the expression of key genes involved in pro-inflammatory pathways [65,70,71,72,73,74].

Glycative stress induced a heterogeneous transcriptional response in the inflammatory panel (Figure 8b). *NF-κB* and *TNFα* expression increased following MGO exposure, whereas IL-6 and p53 showed variable or non-significant changes after correction for multiple comparisons. *TERT* exhibited a modest increase (Figure 8c). Ge-132 alone did not induce a canonical pro-inflammatory transcriptional profile. Under glycative stress, combined Ge-132 + MGO treatment was associated with lower *NF-κB* and *TNFα* expression compared with MGO alone, although these differences were not consistently significant after multiple-comparison correction. IL-6 showed a tendency toward higher expression in the combined treatment group. No significant changes were observed for *p53* across conditions.

Overall, these findings suggest that glycative stress triggers selective transcriptional changes in inflammation-related genes, while Ge-132 may modulate specific components of this response without inducing a coordinated inflammatory transcriptional program. As these experiments were performed exclusively in MEFs, cell type-specific effects cannot be excluded.

## 3. Discussion

The evidence-mapping analysis revealed a fragmented literature, dominated by studies on redox regulation and immune signaling, with glycative stress mechanisms notably underrepresented. Importantly, the evidence-mapping component served primarily as a gap-identification tool rather than a quantitative synthesis or hypothesis generator. It highlighted that glycative had rarely been investigated alongside coordinated cellular responses in a standardized model. These insights directly informed the choice of glycative stress as the experimental context and guided the selection of transcriptional endpoints examined in this study.

The present work was not designed to provide a comprehensive functional characterization of stress-response pathways. Instead, it was conceived as a focused exploratory analysis to contextualize the transcriptional responses associated with the antiglycative effects of Ge-132 under standardized conditions (5 mM Ge-132), chosen based on cytotoxicity data and literature-reported ranges. Consequently, the data do not permit conclusions about functional pathway activation (e.g., *NRF2*, *NF-κB*, glyoxalase, or autophagy), which also depend on protein abundance, subcellular localization, and post-translational modifications. Future studies will require dedicated protein-level and functional assays.

Furthermore, the gene panel, although selected based on established roles in Ge-132-related pathways, represents a targeted subset of transcriptional markers and may not capture the full breadth of cellular responses under glycative stress conditions.

Several reports (Table 1 and Supplementary Table S1) have described redox and anti-inflammatory effects of Ge-132 across a wide range of biological systems. However, the field still lacks a unified experimental framework with standardized conditions. In the present study, we re-evaluated these proposed molecular targets in a single cellular model under controlled glycative stress. This revealed selective, context-dependent transcriptional modulation rather than a broad, coordinated transcriptional response across stress-related pathways. Importantly, the clear reduction in AGE accumulation was accompanied by only limited transcriptional changes under the specific experimental framework. These findings do not allow definitive conclusions about the mechanistic link between reduced glycative damage and downstream cellular responses. Nevertheless, our approach helps bridge previously disconnected biochemical and transcriptional observations.

**Figure 9:**
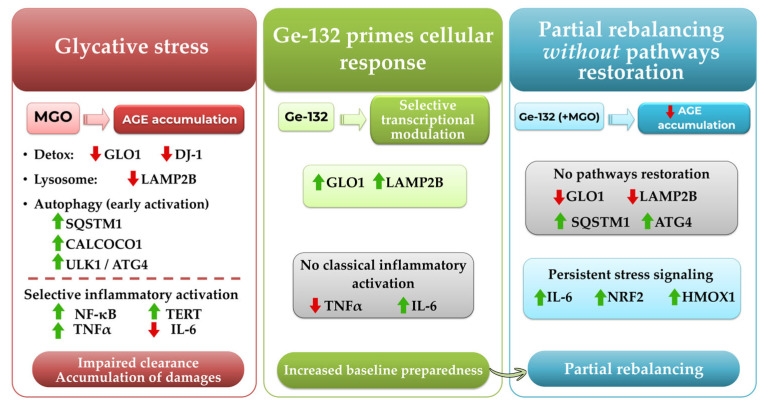
Proposed model of Ge-132 activity under glycative stress. Methylglyoxal (MGO) induces intracellular AGE accumulation along with selective transcriptional responses in stress-related pathways. Ge-132 effectively reduces AGE accumulation while preserving selective transcriptional modulation of detoxification, autophagy, lysosomal, and inflammatory pathways. In this model, the transcriptional responses triggered by glycative stress do not scale proportionally with the biochemical reduction in glycative damage, indicating limited coordination between the biochemical and cellular transcriptional responses under the conditions tested.

The integration of biochemical and transcriptional data supports the following model: glycative stress triggers selective transcriptional modulation of specific stress-response pathways, but these changes do not correspond proportionally to the reduction in glycative damage seen at the protein level (Figure 9). Specifically, exposure to MGO increased the accumulation of AGEs-modified proteins (Figure 6b) while decreasing the expression of key detoxification genes, including the glyoxalase components *GLO1* and *DJ-1*, and lysosomal-associated machinery, such as *LAMP2B* (Figure 8a). These results suggest that glycative stress not only increases the burden of damaged biomolecules but also impairs the cellular systems responsible for clearing them. As a result, damage accumulation and impaired resolution occur together.

In parallel, glycative stress induced selective transcriptional activation of autophagy-related genes, mainly at the early stages of cargo recognition and pathway initiation. The upregulation of *SQSTM1* and *CALCOCO1*, along with increased expression of *ULK1* and *ATG4*, is consistent with an early autophagy response (Figure 7 and Figure S1). However, genes involved in later stages of autophagosome formation showed no significant changes, suggesting that the transcriptional response may be incomplete or not fully coordinated (Supplementary Figure S1).

The inflammatory genes showed a similarly heterogeneous pattern. While *NF-κB* and *TNFα* expression increased under glycative stress, other markers, such as *IL-6* or *p53,* did not show consistent changes. Overall, the transcriptional profiles indicate a differential balance between inflammatory and antioxidant pathways (Figure 8). Under glycative stress alone, *NF-κB* expression rose while *NRF2* induction remained limited. In contrast, the combination of Ge-132 and MGO led to a modest but significant increase in *NRF2* expression. Since NRF2 activity is mainly regulated post-transcriptionally via the KEAP1 system, these results reflect changes in *NRF2*-related transcription rather than direct pathway activation. This pattern aligns with known interactions between *NF-κB* and NRF2 signaling, where the NF-κB subunit p65 can influence *NRF2* transcription in a context-dependent way [75,76]. Although not directly tested here, this interaction offers a useful framework for interpreting the partial and non-coordinated stress responses observed.

Within this framework, Ge-132 shows context-dependent modulatory effects. Under basal (non-stressed) conditions, it increases the expression of selected detoxification and lysosomal genes, including *GLO1* and *LAMP2B*. Under glycative stress, Ge-132 effectively reduces intracellular AGE accumulation at the protein level (Figure 6b). However, it does not restore the MGO-induced suppression of *GLO1, DJ-1*, and *LAMP2B*.

Notably, *NRF2*-related transcriptional activity persisted despite the reduction in AGEs observed (Figure 6b and Figure 7a), further indicating that biochemical protection and transcriptional responses are not directly coordinated.

In addition, glycative stress affected inflammatory signaling in a selective and non-synchronized manner. MGO increased *NF-κB* and *TNFα* expression, while reducing *IL-6* levels (Figure 8b). This divergent pattern suggests that glycative stress does not induce classical pro-inflammatory response, but rather an asymmetric activation of specific signaling components. In this setting, Ge-132 fine-tunes inflammatory signaling in a context-dependent manner rather than simply suppressing it.

Importantly, Ge-132 does not restore the transcriptional downregulation of autophagy, detoxification, or lysosome-related pathways induced by MGO (Figure 7 and Figure 8). Instead of fully restoring cellular homeostasis, Ge-132 appears to rebalance the system primarily at the biochemical level by reducing glycative damage, while stress-response pathways remain transcriptionally active. These findings must be interpreted within the scope of the present study, which examined biochemical and transcriptional responses in a standardized cellular model rather than providing a complete functional characterization of all downstream pathways. Therefore, the observed mRNA expression patterns should be interpreted as indicators of selective transcriptional responses under the tested conditions, not as definitive proof of functional pathway activation.

Overall, our results support a model in which glycative stress leads to the accumulation of damaged biomolecules, reduced expression of detoxification and lysosomal genes, limited transcriptional responses of autophagy-related genes, and dysregulated inflammatory signaling (Figure 9). In this context, Ge-132 reduces glycative damage without fully restoring the associated cellular transcriptional responses, highlighting limited coordination between biochemical protection and adaptive transcriptional programs under the conditions tested.

The biological significance of this observation remains to be established. Importantly, a lack of proportional correspondence between biochemical damage reduction and transcriptional activation of the stress-response pathway is not without precedent. For example, compounds that act through direct chemical trapping of reactive carbonyl species, such as aminoguanidine (the reference inhibitor used in our cell-free assay), reduce AGE formation without inducing transcriptional activation of stress-response pathways. The pattern observed with Ge-132 may reflect a similar upstream mechanism, whereby reduced substrate availability for glycation reactions limits downstream stress signaling without the need for coordinated transcriptional adaptation.

Whether this represents a genuine mechanistic feature of Ge-132 or partly results from the constraints of the present model, including the single time point, single concentration, and MEF-specific context, cannot be resolved from these data alone. Protein-level and functional studies in complementary systems will be required to address this question.

The use of MEFs as a standardized glycative stress model is a key strength of this study. It enabled direct cross-pathway comparison within a single experimental framework and reduced variability associated with cross-model analyses [55,56]. Nevertheless, the observed responses may not generalize to other cell types or in vivo conditions. Future work should therefore extend these findings to more clinically relevant models and systems.

## 4. Materials and Methods

### 4.1. Evidence-Mapping Literature Review: Search and Selection

Article search and selection were conducted in accordance with PRISMA guidelines [77]. The literature search was designed as a multi-step process to retrieve and screen original studies on organic germanium compounds, particularly Ge-132, and their biologically relevant effects on human health. Searches were performed across Web of Science, Scopus, PubMed, Europe PMC, and additional records exported from indexed databases, including ScienceDirect and Embase libraries. The search covered the last 15 years (2011–2025/2026, depending on database availability at the time of retrieval).

Two complementary search strategies were applied. The first, more restrictive approach focused on human-related and clinically relevant studies. It combined terms such as germanium, organic germanium, Ge-132, carboxyethyl germanium, germanium sesquioxide, and carboxyethyl germanium sesquioxide (in title/abstract fields) with human- or patient-related terms. The second, broader approach incorporated biologically relevant contexts, including oxidative stress, antioxidant activity, inflammation, aging, autophagy, glycative stress, therapy, and pharmacological effects, while retaining the core Ge-132-related compound terms. Searches were limited to original articles and, where possible, filtered by biomedical and health-related research areas.

Record deduplication and preliminary filtering were assisted by Julius AI, while final study selection was performed by manual review in accordance with predefined eligibility criteria. Given that the aim of this review was to provide a comprehensive overview of the biological and translational evidence available for Ge-132, we included relevant human, in vitro, and in vivo studies. Studies were considered eligible if the title, abstract, or keywords included at least one Ge-132-related term together with a relevant biological context (e.g., human/patient, clinical, in vitro, in vivo, mouse/mice, or rat).

Eligibility was restricted to original research articles published in the last 15 years that investigated organogermanium compounds, particularly Ge-132 or closely related forms, and reported outcomes relevant to human health or mechanistic biomedical interpretation. These outcomes included oxidative stress, glycative/carbonyl stress, inflammation, autophagy, aging, pharmacological effects, or related cellular responses. We excluded non-original publications, studies focused exclusively on inorganic germanium, non-biomedical applications, agricultural or plant systems, and false-positive records in which “Ge-132” referred to a non-compound identifier. Duplicate records were removed by DOI-based deduplication, followed, when necessary, by title-based normalization. The retained studies were used for qualitative evidence mapping and data extraction, including publication of metadata, experimental models, sample types, dosages, and key biological outcomes.

Due to the heterogeneity of the included literature, the review question was framed using an adapted PICO approach [78]. This focused on the biological effects of organic germanium compounds, particularly Ge-132, across human, animal, and in vitro models, with emphasis on outcomes related to oxidative stress, glycative/carbonyl stress, inflammatory signaling, autophagy, aging, and associated molecular or physiological responses.

#### Risk of Bias Assessment (RoB)

Methodological quality was assessed using an adapted critical appraisal framework based on the Critical Appraisal Skills Programme (CASP) approach, modified to suit the heterogeneous experimental design of the included in vitro and in vivo studies [79]. The assessment evaluated study design clarity, appropriateness of experimental methods, completeness of outcome reporting, potential sources of bias, and transparency of data presentation. For animal studies, additional bias-related domains adapted from preclinical risk-of-bias tools were included, such as allocation procedures, completeness of outcome data, selective reporting, and other potential sources of bias. Data extraction was performed using the Covidence systematic review software [80]. The risk-of-bias assessment covered the following domains: sequence generation, allocation concealment, incomplete outcome data, selective outcome reporting, and other sources of bias. Judgments were categorized as low, high, or unclear risk. Because of the heterogeneity of the included studies, risk-of-bias assessment was conducted separately for in vitro and in vivo studies. This preserved comparability within each evidence stream and supported structured evidence mapping. The domain “other sources of bias” was used to capture potential concerns not covered by the standard domains, including limited independent replication, compound sourcing, and possible funding- or collaboration-related influences.

### 4.2. Cell Culture Conditions

Three cell lines were used in this study: human lens epithelial cells (HLECs), as a model of glycation-associated lens damage; human retinal pigment epithelial cells (ARPE-19), representing retinal tissue vulnerable to oxidative and glycative stress, and mouse embryonic fibroblasts (MEFs), a widely used model for investigating cellular stress responses and mechanisms associated with glycative stress. Unless otherwise specified, cells were cultured in Dulbecco’s Modified Eagle Medium (DMEM, GIBCO) supplemented with 10% fetal bovine serum (FBS), 1% penicillin-streptomycin mix (10,000 U/mL), 1% MEM non-essential amino acids (GIBCO), and 1% sodium pyruvate (100 mM; GIBCO). Cultures were maintained at 37 °C in a humidified incubator containing 5% CO_2_. For treatment experiments, the culture medium was supplemented with Ge-132 at the concentration indicated in each case (Glentham Life Sciences, Corsham, UK, Ref. 7W-GP2575). Ge-132 powder was dissolved in DMEM, the pH was equilibrated to 6.8, and the solution was filtered and used freshly. Methylglyoxal (MGO, Sigma-Aldrich, St. Louis, MO, USA) was diluted in DMEM to a 2 mM final concentration, filter sterilized and then applied to the cells.

### 4.3. Analysis of Cytotoxicity

Mouse embryonic fibroblasts (MEFs), Human Lens Epithelial Cells (HLECs), and human Retinal Pigment Epithelial cells (ARPE19) were kindly provided by E. Bejarano’s group [81]. Cells were generally seeded at a density of 5 × 10^3^ cells per well in 96-well plates and incubated for 24 h. Subsequently, cells were treated with Ge-132 at a concentration ranging from 0 to 20 mM for 24 h. After treatment, cell viability was assessed using the 3-(4,5-dimethylthiazol-2-yl)-2,5-diphenyl tetrazolium bromide (MTT) assay. Briefly, 5 μL of MTT solution (20 mg/mL) was added to each well, followed by incubation at 37 °C for 2 h. Formazan crystals were then solubilized by adding 200 μL of DMSO, and the plate was maintained in the dark at room temperature for 30 min. Absorbance was measured at 570 nm using a Varioskan LUX plate reader (Thermo Fisher Scientific, Vantaa, Finland). Results were analyzed after normalization to the control. All experiments were performed in triplicate.

### 4.4. Quantitative Real-Time PCR Assays

MEFs were cultured in Petri dishes until they reached approximately 80% confluence. Cells were then treated with 5 mM Ge-132 for 24 h, followed by explosion to 2 mM MGO for 2 h. These conditions are consistent with the previously established model for glycative stress [55,81]. Following treatments, cells were washed once with cold 1× PBS, scraped and harvested in the same buffer. After gentle centrifugation, the supernatant was discarded, and the cell pellets were stored at −80 °C until processing. Total RNA was isolated using the NZY Total RNA Kit (NZYTech, Lisboa, Portugal), according to the manufacturer’s instructions. Subsequently, 1 μg of total RNA from each sample was reverse-transcribed using the NZY Reverse Transcriptase Kit (NZYTech, Lisboa, Portugal). Quantitative real-time PCR (qRT-PCR) was performed to evaluate the expression of genes associated with oxidative stress responses, inflammation, glyoxalase-mediated detoxification, cellular stress adaptation, autophagy, and lysosomal pathways. Detailed information on target genes and primer sequences is provided in Supplementary Table S2. PCR reactions were carried out using NZY Speedy qPCR Green Master Mix (NZYTech, Lisboa, Portugal) on a QuantStudio™ 5 Real-Time PCR System. Relative gene expression levels were normalized to the housekeeping gene β-actin and expressed relative to the control group.

### 4.5. In Vitro Antiglycation Assay

The antiglycation activity of Ge-132 was evaluated using a cell-free BSA–glucose model based on a previously described fluorescence assay, with minor adaptations [82]. Briefly, bovine serum albumin (BSA, 10 mg/mL) and anhydrous glucose (90 mg/mL) were prepared separately in phosphate-buffered saline (PBS, pH 7.4). Reaction mixtures were created by combining BSA, glucose, and Ge-132 at the concentrations indicated in the corresponding figure. Aminoguanidine (AG) was included as a positive control for inhibiting AGE formation, and control reactions were prepared in parallel without Ge-132. Sodium azide (0.01%) was added to prevent microbial contamination. The reaction mixtures were incubated at 37 °C in the dark for 7 days. Following incubation, AGE formation was quantified fluorometrically using excitation and emission wavelengths of 360 and 420 nm, respectively. All measurements were performed in triplicate. The percentage inhibition of AGE formation was calculated using the following equation: Inhibition (%) = [(F_0_ − F_1_)/F_0_] × 100, where F_0_ represents the fluorescence intensity of the glycated control and F_1_ the fluorescence intensity of the Ge-132-treated sample.

### 4.6. Protein Purification, Quantification and Western Blot

Protein extracts were prepared in PBS1x buffer supplemented with protease inhibitors (Roche, Complete tablets Ref. 04693132001, Sigma-Aldrich, St. Louis, MO, USA) and quantified using a colorimetric BCA protein assay (Pierce, BCA Protein Assay Kit, REF 23225, Thermo Fisher Scientific, Waltham, MA, USA) according to the manufacturer’s instructions. Samples and standards were analyzed in duplicate, and protein concentration was determined from a standard calibration curve. Protein quantification was performed to ensure equal sample loading for subsequent electrophoretic analyses.

MG-modified proteins were assessed by Western blot. Equal amounts of total protein were mixed with denaturing Laemmli buffer, heat-denatured, and separated by SDS-PAGE. Proteins were subsequently transferred onto a nitrocellulose membrane, which was stained with Ponceau S solution to verify transfer efficiency and equal loading. Membranes were then blocked with 5% (*w*/*v*) non-fat dry milk in TBS-T buffer and incubated overnight at 4 °C with a mouse anti-MG monoclonal antibody (Cell Biolabs, San Diego, CA, USA, ref. STA-011) diluted 1:1000 in TBST-milk 5%. After washing steps, membranes were incubated with the appropriate secondary antibody diluted 1:20,000 for 2 h at room temperature. Immunodetection was performed using the ECL Prime Detection kit (Amersham, UK) and visualized using a digital imaging system (Omega Lum C). Band intensities were quantified by densitometric analysis using ImageJ (Fiji) software [83].

### 4.7. Statistical Analysis and Software

Statistical analyses were performed using GraphPad Prism (version 10.1.2; GraphPad Software, LLC, Boston, MA, USA). Data are presented as mean ± standard deviation (SD) from at least three independent experiments, with the number of technical replicates indicated for each experiment. Outliers among technical replicates were identified by the ROUT method with Q = 0.5%.

For comparisons involving more than two groups, statistical significance was evaluated using one-way analysis of variance (ANOVA) followed by Dunnett’s multiple comparison test, in which each treatment group was compared with the corresponding control. For experiments involving multiple pairwise comparisons against a single control, statistical significance was assessed using an unpaired Student’s *t*-test with Holm–Šidák correction for multiple testing. Differences were considered statistically significant at a *p*-value < 0.05. Statistical significance is indicated as follows: * *p* < 0.05, ** *p* < 0.01, *** *p* < 0.001, and **** *p* < 0.0001.

## 5. Conclusions

In conclusion, the present study combines literature mapping with experimental validation to investigate the biological effects of Ge-132 under glycative stress conditions. The evidence-mapping component was designed to identify underexplored research areas and methodological gaps rather than to synthesize mechanistic evidence. This analysis revealed that glycative stress has received limited attention in the Ge-132 literature and that no previous study had simultaneously examined the cellular pathways investigated here under glycative stress conditions. These findings directly informed and justifyed the experimental framework adopted in this work.

Our results confirm that Ge-132 reduces the accumulation of glycative damage at the biochemical level and indicate that its antiglycative activity may be accompanied by selective and limited transcriptional responses. However, this effect was not associated with the restoration of coordinated transcriptional regulations across key pathways involved in detoxification, autophagy, and inflammation. Instead, Ge-132 elicited a selective and context-dependent modulation of gene expression, suggesting that stress-related transcriptional responses persist despite a measurable reduction in glycative damage.

Collectively, these findings suggest a limited correspondence between the attenuation of glycative damage, as reflected by AGEs accumulation, and the transcriptional responses of cellular stress-related pathways under the conditions tested. Although additional validation at the protein and functional levels is needed to further elucidate the underlying mechanisms, the present work provides a conceptual framework for integrating biochemical and cellular perspectives in future investigations of organogermanium compounds.

## Figures and Tables

**Table 1:** Overview of in vitro studies using Ge-132 in clinical research.

Authors	Study Area	Population Model	Dose Exposure
Nakamura et al. 2015 [16]	Oxidative stress, immunity, inflammation	Human Keratinocytes	THGPA 0.25 to 500 mmol/L. THGPA 0, 2.5, and 5 mM for adrenaline; 0, 1, and 10 mM for ATP.
Kim et al. 2015 [21]	Reproductive biology, oxidative stress, apoptosis	Porcine Oocytes	100 mg/mL, 200 mg/mL, and 400 mg/mL Ge-132.
Shimada et al. 2015 [47]	Biochemistry, immunology, diabetes research	In vitro (water solution)	0.5 M for THGPNa
Tezuka et al. 2017 [18]	Oxidative stress, oncology, inflammation	Hepatocytes from Japanese macaque	0, 0.001, 0.01, 0.1, or 1 μM Ge-132
Kim et al. 2017 [48]	Oxidative stress, apoptosis, cell signaling (KEAP1)	Porcine IVF embryos	0, 100, 200, and 400 μg/mL Ge-132
Shimada et al. 2018 [43]	Biochemistry, inflammation, pain management	In vitro (water solution)	THGP: 0–100 mM
Wada et al. 2018 [1]	Oxidative stress, cell proliferation, immunology, oncology	Mammalian ovary cells; Chinese Hamster Ovary (CHO-K1) cells; HeLa cells, SH-SY5Y cells	0–5 mM Ge-132
Filonova et al. 2019 [49]	Antioxidant properties	Mouse different tumor cells; ovary cells	NMR spectroscopy: Ge-132 at 1.3 × 10^−1^ M and 1.3 × 10^−2^ M. Cyclic voltammetry: Ge-132 at 3 × 10^−3^ M, 6 × 10^−3^ M, and 9 × 10^−3^ M
Takeda et al. 2019 [19]	Oxidative stress, cell signaling and inflammatory response	Human dermal fibroblasts (NHDFs)	0, 5.9, 59, 590, and 5900 µM THGP.LC MS/MS analysis: 0, 1, and 10 mM THGP. PI/Hoechst staining: 0, 5.9 mM THGP.
Wang et al. 2020 [50]	Inflammationimmunity, cell signaling (NF-κb and MAPK pathways)	Primary mouse Mammary Epithelial Cells	Ge-132 at 0.5 μg/mL, 1 μg/mL, and 2 μg/mL
Mertens et al. 2020 [29]	Oncology, oxidative stress	MDA-MD-175; MDA-MB-231; MRC5; H460; A2780	Germanium(IV)-diketonat at 5 μM or 10 μM
Baidya et al. 2021 [51]	Immunity, antiviral strategies, inflammation	RAW 264.7; HEK293T; A549; MDCK	THGP at 0, 20, 200, and 2000 μg/mL
Azumi et al. 2022 [26]	Inflammation	Human THP-1 monocytes; mouse RAW264	THGP at 0.05, 0.5, 1.2, 5 and 12 mM; Ge-132 500 mM
Sekiguchi et al. 2023 [52]	Pain management, oxidative stress, calcium channel signaling	Cav3.2-transfected HEK_293 (human kidney)	THGP at 1, 3 and 10 mM
Azumi et al. 2023 [22]	Immunity, oncology	RAW 264.7 mouse macrophage-like cells	500 µM THGP. Dose dependent: THGP from 50 to 5000 µM
Cruz et al. 2024 [30]	Oxidative stress	Bovine Sperm cells	0–500–1000 μg/mL of Ge-132
Takeda et al. 2024 [37]	Immunology, antioxidant, cardiovascular/gastrointestinal health	Mouse cell RAW264.7	0, 50, 500, and 5000 μM THGP

Abbreviations: Ge-132, carboxyethyl germanium sesquioxide; THGP, 3-(trihydroxygermyl)propanoic acid. Study area refers to the primary biological process investigated in each study, as defined by the authors. Population models include in vitro cellular systems. Dose/exposure is reported as described in the original publications.

**Table 2:** Overview of in vivo studies using Ge-132 in clinical research.

Authors	Study Area	Population Model	Dose Exposure
Nakamura et al. 2012 [53]	Immunology, inflammation	BALB/C Cr slc mice	0.0012–0.03% Ge-132, equivalent to 50 mg/kg body weight per day
Nakamura et al. 2012 [25]	Oncology, gastroenterology, microbiology	Wistar rats	0.05% Ge-132/day
Nakamura et al. 2014 [17]	Immunology, oxidative stress, toxicology	ICR mice	0.05% Ge-132 0, 1, and 4 days.
Reddeman et al. 2020 [5]	Toxicology	Han:WIST rats: M610	0, 500, 1000, 2000 mg/kg bw/day Ge-132
Wang et al. 2020 [50]	Inflammation, immunology	BALB/c mice	Ge-132 at 5 mg/kg, 10 mg/kg, and 20 mg/kg
Baidya et al. 2021 [51]	Immunology, virology	MAVS KO and C57BL/6J mice	50 or 100 mg/kg THGP
Sekiguchi et al. 2023 [52]	Cystitis- and pancreatisis-related pain, oxidative stress, calcium signaling	*ddY* mice	THGP 0.1–1 nmol/paw (intraplantar), THGP 10–100 mg/Kg (systemic). Visceral pain: THGP 100 mg/Kg (systemic)
Takeda et al. 2024 [37]	Immunology, hematopoiesis, oxidative stress, inflammation	C57BL/6 J and ICR mice	Diet with 0.05% Ge-132/day

Abbreviations: Ge-132, carboxyethyl germanium sesquioxide; THGP, 3-(trihydroxygermyl)propanoic acid. Study area refers to the primary biological process investigated in each study, as defined by the authors. Population models include in vitro cellular systems. Dose/exposure is reported as described in the original publications.

## Data Availability

The original contributions presented in this study are included in the article. Further inquiries can be directed to the corresponding author.

## Supplementary Materials

The following supporting information can be downloaded at: https://www.mdpi.com/article/10.3390/molecules31142405/s1, Figure S1: Relative expression of autophagy and lysosomal-associated genes. Table S1: Ge-132 evidence-mapping review, Full tables, Table S2: qRT-PCR Oligonucleotides.
