# Supplementary material for: Organic Germanium (Ge-132) Reduces Glycative Damage While Maintaining Cellular Stress Signaling, Revealing Limited Coordination Between Biochemical and Cellular Responses
Source: Molecules. 2026 Jul 8;31(14):2405. doi: 10.3390/molecules31142405 (PMC13413932; doi:10.3390/molecules31142405)
Supplement: Supplementary file 1 [file molecules-31-02405-s001.zip › Figure S1. Relative expression of autophagy and lysosomal-associated genes.pdf]

Supplemental Figure S1

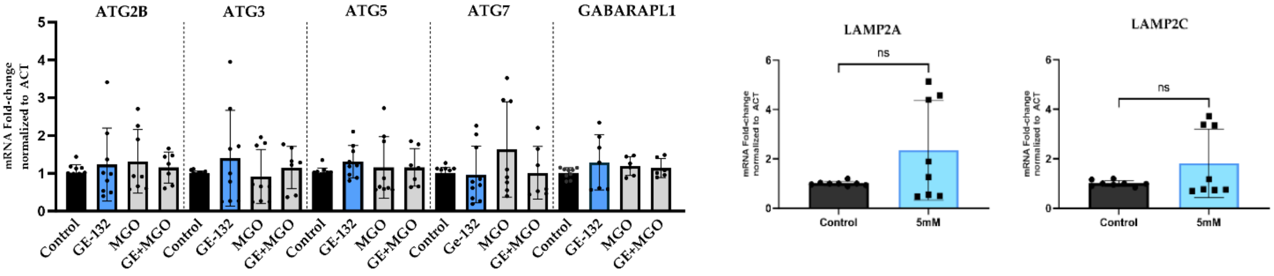


(a) (b)

Figure S1. Relative expression of autophagy and lysosomal-associated genes. (a) MGO induced upregulation of an early regulatory components of autophagy (*ATG4*), whereas genes involved in autophagosome elongation (i.e. *ATG2B, ATG3, ATG5, ATG7*) showed limited or no consistent changes. GABARAPL1, which is mainly involved in autophagosomal membrane expansion, cargo enclosure, and later maturation/fusion steps, is not displaying any change across the treatments. (b) Expression of lysosomal-associated genes LAMP2A and 2C remained unaltered across Ge-132 and MGO treatments. Expression levels were normalized to Actin and are presented relative to control. Data represent mean ± SD from 3 independent experiments and an average of 6–10 technical replicates. Statistical significance indicated as $*p < 0.05$.
