# Supplementary material for: Organic Germanium (Ge-132) Reduces Glycative Damage While Maintaining Cellular Stress Signaling, Revealing Limited Coordination Between Biochemical and Cellular Responses
Source: Molecules. 2026 Jul 8;31(14):2405. doi: 10.3390/molecules31142405 (PMC13413932; doi:10.3390/molecules31142405)
Supplement: Supplementary file 1 [file molecules-31-02405-s001.zip › Table S2. qRT-PCR Oligonucleotides.pdf]

Supplemental Table S2

ANTIOXIDANT	*NFE2L2 (NRF2)*	ATGACAATGAGGTTTCTTCGG	FW
		CAATGAAGACTGGGCTCTC	REV
	HMOX1	AACTCCCTGGAGATGACTC	FW
		CTCAAAGAGCTGGATGTTGAG	REV
	GCLM	GTTGACATGGCCTGTTCAG	FW
		AACTCCATCTTCAATAGGAGGT	REV
	GCLC	AAGTGGATGTGGACACCAG	FW
		CTGTCATTAGTTCTCCAGATGC	REV
	NQO1	ACATCACAGGTAAACTGAAGG	FW
		TCAGATGGCCTTCTTTATAAGC	REV
MACROAUTOPHAGY	*SQSTM1*	CTGGGACTGAGAAGGCTCAC	FW
		GCAGCTGATGGTTTGGAAAT	REV
	NDPS2 (CALCOCO2)	ACCATGGAGGAGACCATCAA	FW
		TTCTGGACGGAATTGGAAAG	REV
	ULK1	TCATCTTCAGCCACGCTGT	FW
		CACGGTGCTGGAACATCTC	REV
	ATG2B	AACTCACAAACAGAATGGTTCAAA	FW
		AAGGGTACCAGGAAGACACCA	REV
	ATG3	CATGCAGGCATGCTGAGGTG	FW
		CGTTAACAGCCATTTTGCCACT	REV
	ATG4D	CACATCCTCAGGAAAGCCGT	FW
		GACCACAGACTTCCACTCGG	REV
	ATG5	GGGAAGCAGAACCATACTATTG	FW
		AAATGTACTGTGATGTTCCAAGG	REV
	ATG7	AGGAGATTCAACCAGAGACC	FW
		GCACAAGCCCAAGAGAGG	REV
	GABARAPL1	ACCATGGGCCAACTGTATGA	FW
		TGGGCTTCCAACCACTCATTT	REV
LYSOSOMES	*LAMP2A*	GTGCAACAAAGAGCAGACTGT	FW
		GGCACAAGGAAGTTGTCGTC	REV
	LAMP2B	AGAGTGTTCGCTGGATGATG	FW
		TGCCAATTACGTAAGCAATCA	REV
	LAMP2C	AAGGGTTCAGCCTTTCAATG	FW
		ACAATTATAAGGAAGCCCAAGG	REV
INFLAMMATION	*TP53 (p53)*	CCTCAGCATCTTATCCGAGTGG	FW
		TGGTGAGGATGGGCCTCC	REV
	IL-6	AAATTCGGTACATCCTCGACG	FW
		TTTCACCAGGCAAGTCTCC	REV
	TNF (TNF- α*)*	CTCTTCTGCCTGCTGCACTTTG	FW
		CCACTGGAGCTGCCCCTCAG	REV
	TERT	AGAGTGTCTGGAGCAAGTTG	FW
		AGTCCATGTTCACAATCGGC	REV
	NF- κb	GAACCACACCCCTGCATATAG	FW
		GCACAACTCCTTCATCCTCTC	REV
PREGLYCATION	*GLO1*	ATTGCCGTTCCTGATGTCTAC	FW
		GAATCTCAATCCAGTAGCCGTC	REV
	GLO2	TGGGCTGAAGGTTTATGGAG	FW
		CAGATGTGTCCCGAAGTATGG	REV
	PARK7 (DJ-1)	ATTTATCTGAGTCGCCTATGGTG	FW
		GCATCCAAAACCTACTTCGTG	REV
Housekeeping	β*–ACTIN*	GTACGACCAGAGGCATACAG	REV
		ACCGTGAAAAGATGACCCAG	FW
